# Electrophysiological correlates of masked repetition and conceptual priming for visual objects

**DOI:** 10.1002/brb3.1415

**Published:** 2019-09-26

**Authors:** Bingbing Li, Chuanji Gao, Juan Wang

**Affiliations:** ^1^ School of Education Science Jiangsu Normal University Xuzhou China; ^2^ Department of Psychology Institute for Mind and Brain University of South Carolina Columbia SC USA

**Keywords:** masked conceptual priming, masked repetition priming, N/P190, N400, visual object processing

## Abstract

**Background:**

Previous studies have investigated the time course of visual object processing using event‐related potential (ERP) and the masked repetition priming paradigm. However, it is unclear how the ERP correlates associated with masked repetition priming differentiate from masked conceptual priming of visual objects.

**Method:**

The present study used semantically related picture pairs of visual objects to compare the ERPs associated with masked repetition and conceptual priming of visual objects.

**Results:**

The results revealed that masked repetition priming was associated with N/P190 and N400 effects, whereas masked conceptual priming was only associated with N400 effect. Moreover, the topography of repetition N/P190 effect was different from repetition and conceptual N400 effects, whereas the topography of repetition N400 effect was similar to conceptual N400 effect.

**Conclusions:**

These results indicated that masked repetition and conceptual priming were associated with spatiotemporally different ERP effects and that the N400 of visual objects was sensitive to automatic semantic spreading.

## Graphical abstract

We compared the event‐related potentials associated with masked repetition and conceptual priming for visual objects. The results revealed that masked repetition priming of visual objects was associated with N/P190 and N400 effects. Masked conceptual priming of visual objects was associated with N400 effect. N/P190 reflects perceptual processing, and N400 reflects semantic processing.

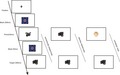

## 1 INTRODUCTION

Masked priming paradigm is one of the most widely used tools to investigate visual stimulus processing (e.g., words and objects). In a typical masked priming experiment, a masked prime item is briefly presented (e.g., 50 ms) prior to the target item. Subjects respond faster and more accurately to the target when the prime is the same (repetition priming) or semantically related to the target (conceptual priming) than when they are unrelated (Forster & Davis, 1984; Forster, Mohan, Hector, Kinoshita, & Lupker, 2003; Grainger & Holcomb, 2009). Subjects are usually unaware of the prime or unable to identify it in masked priming experiments, which prevents higher level processing of the prime (e.g., strategic or conscious processing). Therefore, the masked priming effects were assumed to reflect automatic visual processing (Forster et al., 2003).

Over the last decades, numerous studies have combined the masked priming paradigm with event‐related potential (ERP) technique, which has high temporal resolution, to investigate the time course of visual processing. Although most of these studies focused on the time course of letter or word processing (Brown & Hagoort, 1993; Grainger & Holcomb, 2009; Holcomb & Grainger, 2006; Misra & Holcomb, 2003), the time course of visual object processing was also investigated by some studies in the last decade, using the masked repetition priming paradigm and ERP (Eddy & Holcomb, 2009, 2010, 2011; Eddy, Schmid, & Holcomb, 2006). It was found that an earlier anterior N/P190, which is likely associated with earlier perceptual processing, an anterior N300, which might reflect object‐specific representations processing, and a widely distributed N400, which might reflect semantic processing, were associated with masked repetition priming effects. However, three questions regarding the time course of visual object processing remain unresolved.

First, it is unclear whether semantic processing of visual objects occurs at an early temporal stage. Although previous studies suggested that N/P190 was associated with facilitated early perceptual processing of visual objects (e.g., Eddy et al., 2006), some studies found that the magnitude of N/P190 effect was not modulated by some perceptual properties such as orientation change of the prime (Eddy & Holcomb, 2009, 2010, 2011), indicating a remaining possibility of its association with higher level cognitive processing. In addition, previous studies mainly used masked repetition priming, which has difficulty of dissociating perceptual processing from semantic processing. Thus, it is worthwhile to use masked conceptual priming paradigm to test the role of the N/P190, wherein the prime is semantically related (e.g., HORSE vs. cow) but not perceptually same to the target.

Second, it is still an open question whether the N300 was related to perceptual, conceptual, or object‐specific processes. Research showed that the N300 priming effect was observed in studies of unmasked repetition or semantic priming paradigm when picture stimuli were used. But similar effects were not reported using word stimuli (e.g., Barrett & Rugg, 1989; Barrett & Rugg, 1990; Holcomb & McPherson, 1994; McPherson & Holcomb, 1999). These findings suggested that the N300 unmasked priming effect might reflect more efficient object‐specific processing. However, it is not clear whether the N300 masked priming effect for visual objects would be influenced by perceptual processing or semantic processing with the current stage of knowledge. The comparison between ERPs associated with masked repetition and conceptual priming, which could contribute to dissociate perceptual from conceptual processing, is suitable to examine the underlying mechanism of the N300.

Third, further evidence was needed about whether the N400 priming effect was indeed associated with automatic semantic processing. Previous studies have found that unmasked conceptual priming for visual objects was associated with the late N400 effect (Chauncey, Holcomb, & Grainger, 2009; Kovalenko, Chaumon, & Busch, 2012; McPherson & Holcomb, 1999). Modulation of the N400 priming effect was also found using masked repetition priming paradigm (e.g., Eddy et al., 2006). However, it is unclear whether the N400 effect is related to automatic semantic processing of the picture stimuli or interactions between form and meaning because masked repetition priming could induce both processes (e.g., Eddy et al., 2006; Grainger & Holcomb, 2008). Thus, the specific function of the N400 priming effect could be determined by examining whether masked conceptual priming can induce similar N400 effect.

Therefore, although previous studies have provided insights about the time course of visual object processing by examining the ERPs associated with masked repetition priming, more evidence is clearly needed. Measuring ERPs to masked conceptually but not perceptually similar objects will contribute to our understanding of specific perceptual and conceptual cognitive processes occurring in different temporal processing stages. First, it would provide evidence about whether conceptual processing can occur at early temporal stage. Second, it helps to evaluate whether the N300 also reflects perceptual or conceptual processes. Finally, it would help to test whether the N400 effect reflects automatic semantic processing.

The present study compared the ERP effects associated with masked repetition and conceptual priming of visual objects to investigate the time course of visual object processing, using semantically related picture pairs of common objects as stimuli. We predicted that subjects should respond faster in the related condition compared with unrelated condition in both repetition and conceptual priming blocks as suggested by previous behavioral studies using masked repetition and conceptual priming paradigm (e.g., Dell'Acqua & Grainger, 1999). We predicted that masked repetition priming of visual objects would be associated with N/P190, N300, and N400 effects, whereas masked conceptual priming of visual objects would be associated with the N400 effect based on previous studies (e.g., McPherson & Holcomb, 1999).

## 2 METHOD

### 2.1 Participants

Twenty‐four right‐handed students (age 19–21 years, nine males) from Jiangsu Normal University took part in the present experiment. All of them had normal (or correct to normal) vision. Data from one participant were excluded from both behavior and ERP analysis because of excessive EEG artifacts (>30%), leaving a final sample of 23 participants (age 19–21 years, eight males). The experiment protocol was approved by the Human Research Ethics Committee of Jiangsu Normal University. All subjects gave written informed consent and were paid for their participation.

### 2.2 Stimuli

Stimuli were 240 pairs of semantically related color pictures of common objects with white background. For the selection of the pictures, 158 of the picture pairs were from the POPORO picture database (Kovalenko et al., 2012). Eighty‐two of the picture pairs were developed from Internet sources with the same procedure described in the POPORO paper. Thus, all images have the same properties such as size and background color. Another 40 pictures were used in practice block. The mask stimulus was a kaleidoscope picture from Voss, Baym, and Paller (2008).

The experiment consisted of two blocks: one repetition priming and one conceptual priming block. In the repetition priming block, target was preceded by either the same item (identity‐related condition) or a semantically unrelated item (unrelated condition). In the conceptual priming block, target was preceded by either a semantically related item (semantically related condition) or a semantically unrelated item (unrelated condition). A total of 120 trials (half related and half unrelated trials) were presented randomly in each block. Target pictures were equally subdivided into four sets. For each set, four prime‐target pair lists corresponding to four different experimental conditions were created: one for identity‐related condition in the repetition priming block (the prime was the same picture as the target), one for unrelated condition in the repetition priming block (the prime was another semantically unrelated target picture in the same set), one for semantically related condition in the conceptual priming block (the prime was the corresponding semantically related prime picture), and one for unrelated condition in the conceptual priming block (the prime was another semantically unrelated prime picture in the same set). The assignment of the four sets to different experimental conditions was counterbalanced across subjects. The order of the blocks was also counterbalanced across participants.

### 2.3 Procedure

**Figure 1:**
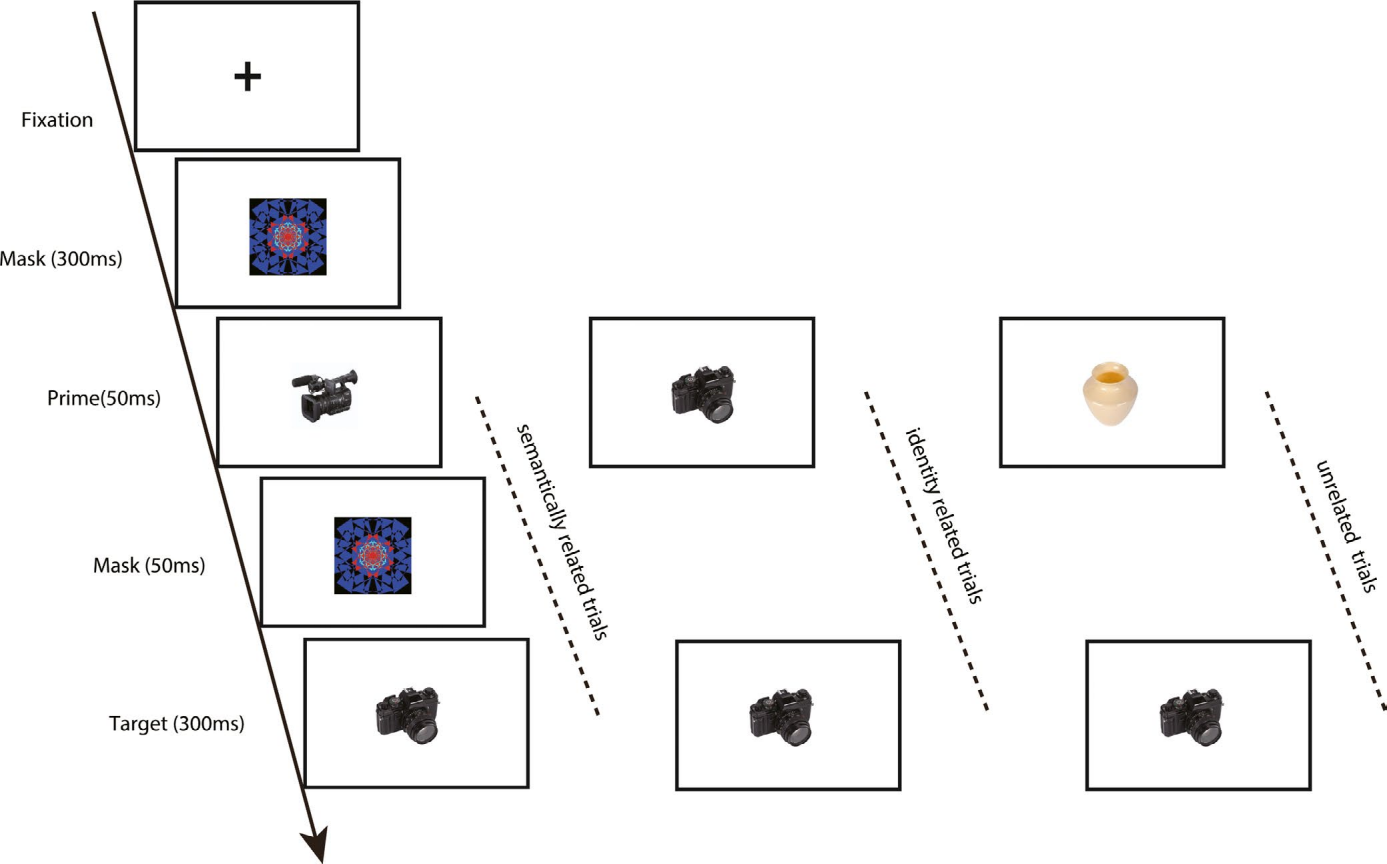
The schematic of the trial in the experiment

The presentation of a trial in the experiment was shown in Figure 1. Stimuli were presented on a screen with white background about 75 cm from the subjects. Each trial began with a forward mask for 300 ms, followed by a prime for 50 ms, then a postmask for 50 ms and the target for 300 ms. The ISI between trials was randomized between 800 and 1,300 ms. Participants were asked to indicate whether the target was interesting or not (i.e., whether they thought the object in the picture was funny, pleasant, or was attractive) with two buttons. In order to encourage semantic processing of the images, we asked the subjects not to do the task based on superficial features of the objects and make decision after they get the semantic meaning of the objects. A practice block was administered before the formal experiment to make participants adapt to the experiment procedure.

### 2.4 EEG recording and data analysis

EEGs were recorded by 64 electrodes embedded in a cap using Brain Products system with a sample rate of 1,000 Hz. The band pass was 0.01–100 Hz (0.01–40 Hz filtered offline). All electrodes were referenced to left mastoid online and re‐referenced to the mean of left and right mastoid in offline analysis. Four additional electrodes were placed above and below the left eye and on the left and right canthi to record the vertical and horizontal EOG. The impedance was kept below 5 kΩ during the recording. EEGs were cut into 850 ms epochs with −200 ms prior and 650 ms after the target item (the first 100 ms was served for baseline correction). EOG and muscle artifacts were corrected using ICA method. Epochs that exceeded 75 μV or −75 μV (6.8% trials were excluded), exceeded 100 μV within a 200 ms moving time window (2% trials were excluded), or contained activities below 0.5 μV for over 200ms (0.2% trials were excluded) were excluded. The offline analysis was conducted using EEGlab (Delorme & Makeig, 2004) and ERPlab (Lopez‐Calderon & Luck, 2014) MATLAB toolboxes.

For the analysis of behavioral data, a two‐way repeated measure ANOVA (RM‐ANOVA, criterion *p* < .05) with priming type (repetition priming/conceptual priming) and trial congruency (related/unrelated) was conducted on the mean response times (RTs) to the target stimuli in related and unrelated condition (RTs which exceeded three standard deviations were omitted from averaging). The time windows and electrode selection for the analysis of ERP data were based on previous studies (Eddy & Holcomb, 2010). Three time windows, 100–250 ms for N/P190, 250–350 ms for N300, and 350–500 ms for N400, were selected. Nine electrode clusters were selected: left frontal electrodes (LF): AF3, F3; middle frontal electrodes (MF): AFz, Fz; right frontal electrodes (RF): AF4, F4; left central electrodes (LC): FC3, C3; middle central electrodes (MC): FCz, Cz; right central electrodes (RC): FC4, C4; left parietal electrodes (LP): CP3, P3; middle parietal electrodes (MP): CPz, Pz; and right parietal electrodes (RP): CP4, P4. Four‐way RM‐ANOVA, with prime type (repetition priming/conceptual priming), trial congruency (related/unrelated), location (anterior, central, posterior), and hemisphere (left, middle, right) as factors, was conducted separately for each time window for the analysis of ERP data. Greenhouse–Geisser correction was applied where appropriate. However, uncorrected degree of freedom with corrected *p* value was reported.

## 3 RESULTS

### 3.1 Behavioral results

The results of the two‐way ANOVA revealed a significant two‐way interaction between priming type and trial congruency, *F* (1, 22) = 5.73* p* = .026, *η* ^2^ = 0.21. Participants responded faster in related condition compared with unrelated condition in repetition priming block, 520 (*SE*: 36) ms versus 535 (38) ms, *t* (22) = 2.69, *p* = .013, *d* = 0.56, but not in conceptual priming block, 538 (38) ms versus 539 (39) ms, *t* (22) = 0.102, *p* = .921, *d* = 0.02.

### 3.2 ERP results

**Figure 2:**
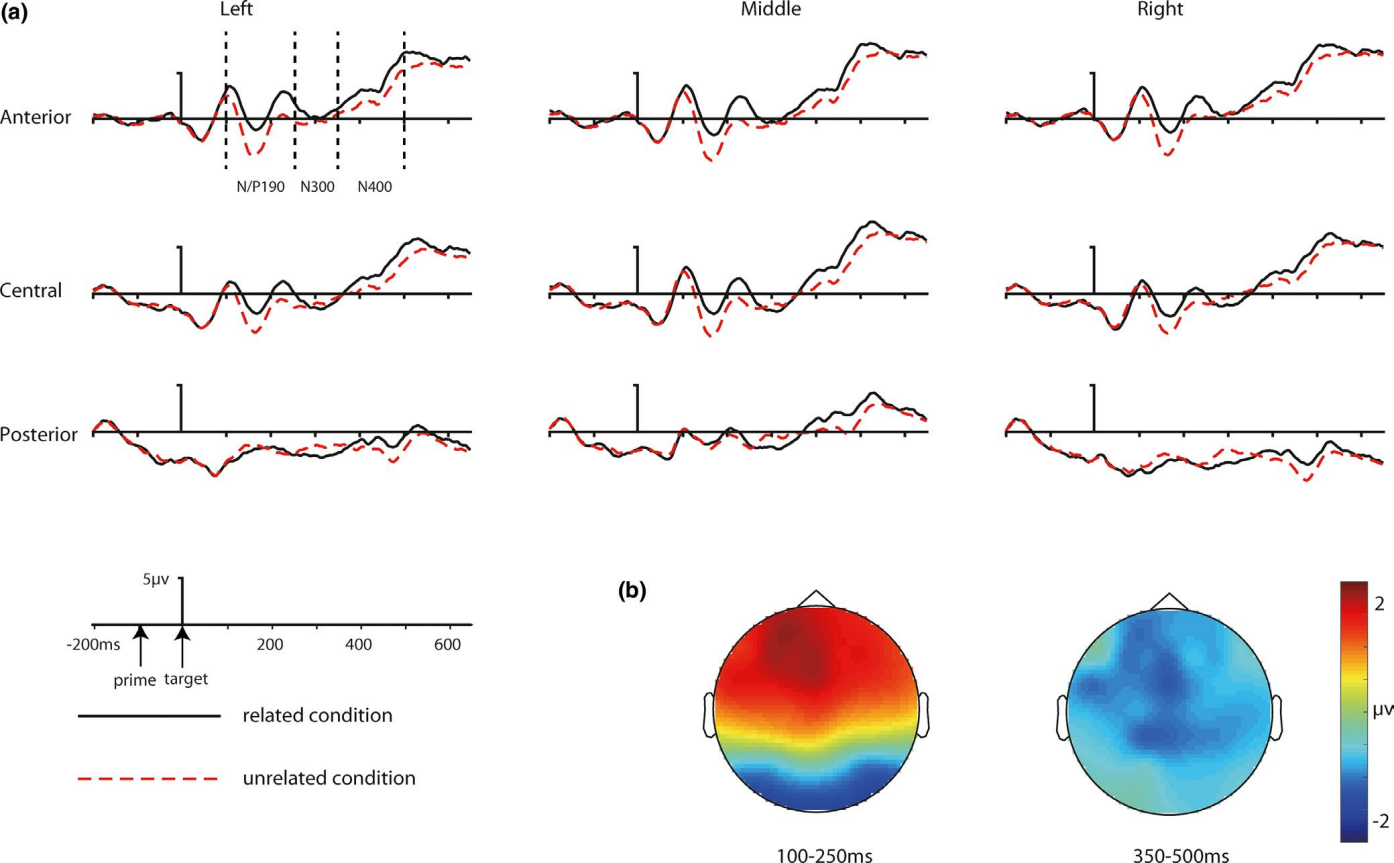
The grand‐averaged ERP waveforms and the topography maps for masked repetition priming. (a) ERP waveforms of related and unrelated condition. From top to bottom: electrode cluster of anterior, central, and posterior region; from left to right: electrode cluster of left, middle, and right hemisphere. (b) Topography maps for grand‐averaged difference between related and unrelated condition at 100–250 ms (N/P190, ERPs to related target minus ERPs to unrelated target) and 350–500 ms (N400, ERPs to unrelated target minus ERPs to related target)

**Figure 3:**
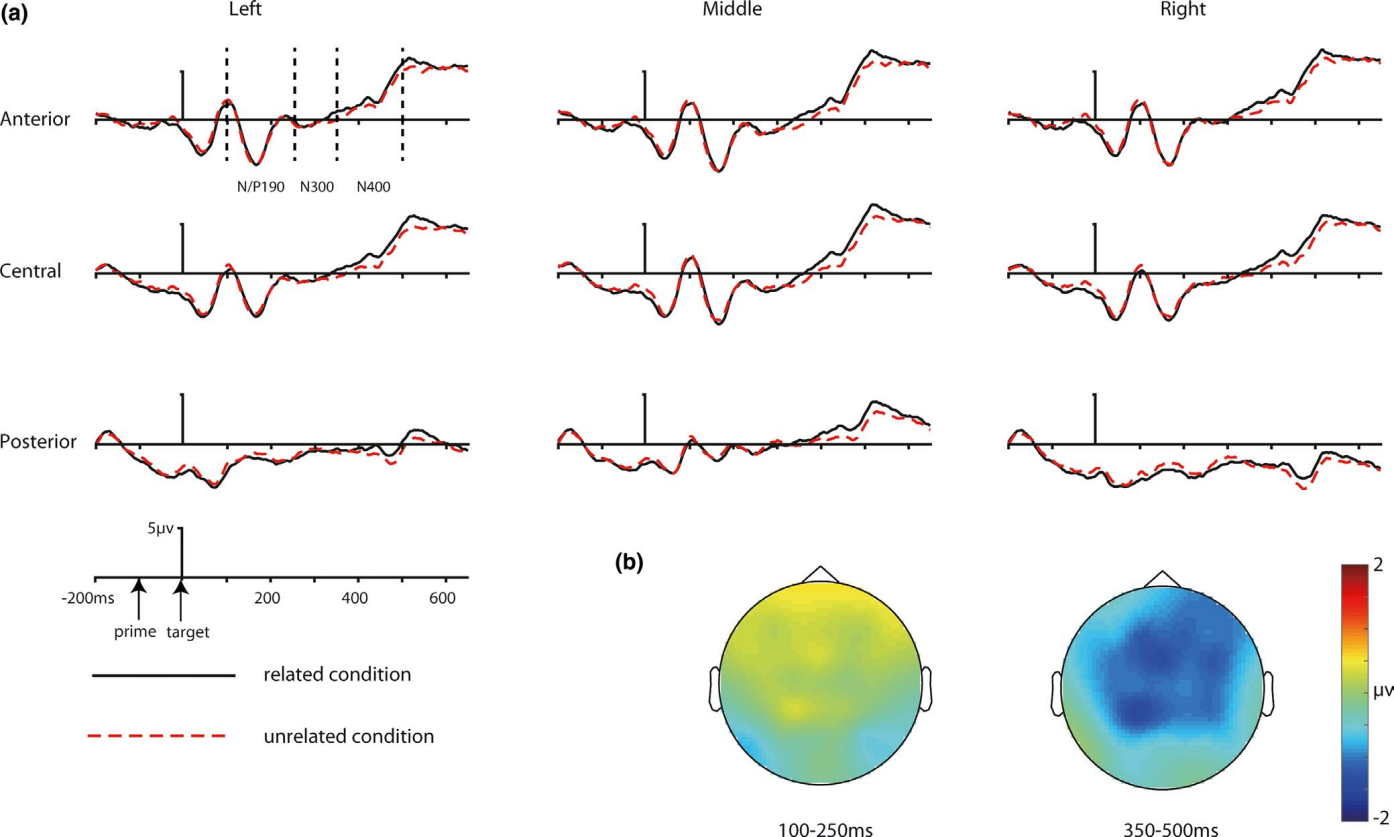
The grand‐averaged ERP waveforms and the topography maps for masked conceptual priming. (a) ERP waveforms of related and unrelated condition. From top to bottom: electrode cluster of anterior, central, and posterior region; from left to right: electrode cluster of left, middle, and right hemisphere. (b) Topography maps for grand‐averaged difference between related and unrelated condition at 100–250 ms (N/P190, ERPs to related target minus ERPs to unrelated target) and 350–500 ms (N400, ERPs to unrelated target minus ERPs to related target)

The grand‐averaged ERP waveforms and the topography of N/P190 and N400 priming effects were shown in Figure 2 for repetition priming and Figure 3 for conceptual priming. Mean numbers of artifact‐free trials for related and unrelated conditions for repetition priming block were 54 (±8) and 54 (±9). Mean numbers of artifact‐free trials for related and unrelated conditions for conceptual priming block were 53 (±10) and 53 (±10).

#### 3.2.1 100–250 ms (N/P190)

In line with previous studies (Eddy et al., 2006), the polarity of the N/P190 component is negative at anterior but positive at posterior electrodes. The four‐way interaction effect of the four‐way RM‐ANOVA was not significant, *F* (4, 88) = 0.67,* p* = .634, *η* ^2^ = 0.03. The three‐way interaction involving prime type, trial congruency, and location was significant, *F* (2, 44) = 12.27,* p* = .001, *η* ^2^ = 0.36. The ERP amplitudes in related condition were less negative compared to unrelated condition at frontal, *t* (22) = 8.06, *p* < .001,* d* = 1.68 and central electrodes, *t* (22) = 6.04, *p* < .001,* d* = 1.26, but not at parietal electrodes, *t* (22) = 0.3, *p* = .768, *d* = 0.06 for repetition priming, whereas the amplitudes of ERPs in related condition were not different from unrelated condition at all the three electrode locations for conceptual priming (all *p* > .1). Other three‐way interaction effects involving trial congruency were not significant (all *p* > .05).

#### 3.2.2 250–350 ms (N300)

The four‐way interaction of the four‐way RM‐ANOVA was not significant, *F* (4, 88) = 0.61, *p* = .617, *η* ^2^ = 0.03. None of the three‐way interaction effects were significant (all *p* > .05). The two‐way interaction between trial congruency and location was significant, *F* (2, 44) = 11.99,* p* = .001, *η* ^2^ = 0.35. The ERP amplitudes in related condition were more negative compared with unrelated condition at parietal electrodes, *t* (22) = 2.25, *p* = .035,* d* = 0.47, but not at central, *t* (22) = 0.26, *p* = .801,* d* = 0.05, and frontal electrodes, *t* (22) = 1.23, *p* = .232, *d* = 0.26. However, this result was not consistent with a typical priming effect (ERPs should be less negative in related condition than unrelated condition), which was also reported in previous studies (Eddy & Holcomb, 2009, 2010). None of other two‐way interactions involving trail congruency was significant (all *p* > .05).

#### 3.2.3 350–500 ms (N400)

None of the four‐way interaction effects, three‐way interaction effects, or two‐way interaction effects involving trail congruency was significant (all *p* > .05). The four‐way RM‐ANOVA only revealed a significant effect of trial congruency, *F* (1, 22) = 9.86* p* = .005, *η* ^2^ = 0.31. The ERP amplitudes in unrelated condition were more negative compared with related condition at frontal, *t* (22) = 2.95 *p* = .007,* d* = 0.62, central, *t* (22) = 3.06, *p* = .006,* d* = 0.64, and parietal electrodes, *t* (22) = 2.94, *p* = .008, *d* = 0.62.

#### 3.2.4 Topographic comparison

Topographic comparison was performed to explore whether the topographic distribution of the priming effects was different, using the vector scale method of McCarthy and Wood (1985). A significant interaction between comparison and electrode would indicate that the two ERP effects have different topography. The comparison on the repetition N/P190 effect and the repetition N300 effect revealed that the two priming effects had a different topographic distribution, *F* (61, 1,342) = 17.61, *p* < .001, $\eta_{p}^{2}$ = 0.45. The comparison on the repetition N/P190 effect and the repetition N400 effect revealed that the two priming effects had a different topographic distribution, *F* (61, 1,342) = 12.52, *p* < .001, $\eta_{p}^{2}$ = 0.36. The comparison on the repetition N300 effect and the conceptual N300 effect revealed that the topographic distribution of the two priming effects was not significantly different, *F* (61, 1,342) = 1.33, *p* = .25, $\eta_{p}^{2}$ = 0.06. The comparison on the repetition N400 and the conceptual N400 effect revealed that the topographic distributions of the two priming effects were not significantly different, *F* (61, 1,342) = 0.69, *p* = .722, $\eta_{p}^{2}$ = 0.03. The comparison on the conceptual N300 effect and the conceptual N400 effect revealed that the two priming effects had a different topographic distribution, *F* (61, 1,342) = 2.27, *p* = .04, $\eta_{p}^{2}$ = 0.09.

## 4 DISCUSSION

The present study compared ERPs associated with masked repetition and conceptual priming of visual objects using semantically related picture pairs to explore the time course of visual object processing. The results indicated that masked repetition and conceptual priming of visual objects were associated with spatiotemporally different ERP components, which suggested that the two types of priming are associated with different time course.

The behavioral priming effect was significant for repetition priming but not for conceptual priming. These results suggested that the RTs in the present study were affected by the perceptual overlap between the prime and target in the repetition block but not by the semantic overlap between the prime and target in the conceptual priming block. The nonsignificant conceptual priming effect was on contrary to our prediction and was inconsistent with previous studies that had found significant behavioral masked conceptual priming effect for picture stimuli (e.g., Dell'Acqua & Grainger, 1999; Van den Bussche, Van den Noortgate, & Reynvoet, 2009). We suspect that this discrepancy was because of different task used across studies. Most previous studies used semantic category judgment task compared with interesting/noninteresting judgment in the present study. Although we encouraged subjects to make semantic processing of the target, the task in the present study might not be as sensitive to masked conceptual priming as semantic category judgment task. However, the significant N400 conceptual priming effect indeed suggested that the semantic processing of the target was affected by conceptual priming.

In line with our expectation, the N/P190 effect was significant in the repetition priming block but not in the conceptual priming block. The topography comparison between the N/P190 and the repetition and conceptual N300 and N400 priming effects suggested that the N/P190 effect was spatiotemporally different from the N300 and N400 effects, which indicated that they reflect different stages of visual object processing. The comparison between masked repetition priming and conceptual priming can isolate the perceptual processing from the semantic processing without contamination of strategic factors. Therefore, these results provided stronger evidence for the hypothesis that the N/P190 effect reflected facilitated early perceptual processing of the target induced by superficial overlap between the prime and target (Eddy & Holcomb, 2009, 2010, 2011; Eddy et al., 2006), and conceptual processing of visual objects might not occur at an early temporal stage.

Similar N300 effect (in magnitude and topographic distribution) was observed in the repetition priming and conceptual priming blocks. The topographic comparison revealed that the distribution of the N300 effect was different from the repetition N/P190, and the repetition/conceptual N400 effects, which suggested that the N300 effect was a functionally different ERP component compared with the N/P190 and the N400. Given the similarities and differences between our study and previous studies, and the similar topographic distribution of the repetition and conceptual N300 priming effects, our findings suggested that this effect was not altered by the use of a different paradigm and was not likely to be a perceptual or semantic effect. Instead, our findings supported the proposal that it was related to object‐specific representation processing (Eddy et al., 2006). However, the N300 effect in the present study was reversed compared with previous studies. A similar reversed N300 effect was also reported by previous studies (Eddy & Holcomb, 2010). One possible explanation for the reversed N300 effect is that the N300 effect tends to merge with the N400 effect or be overlapped by the N/P190 effect with short prime duration or short SOA between the prime and target (Eddy & Holcomb, 2009, 2010). The reversed N300 effect should not be caused by unsuccessful experimental control because similar experimental procedure (e.g., prime duration, SOA) was used in the present study compared with the original study that found typical N300 effect (Eddy et al., 2006).

Similar N400 effect (in magnitude and topographic distribution) was observed in the repetition priming and conceptual priming blocks. These results suggested that the masked repetition and conceptual N400 priming effect might reflect similar processing and that the N400 masked repetition priming effect obtained in previous studies did reflect facilitated sematic processing of visual objects (Eddy et al., 2006; Kutas & Federmeier, 2011). Our results were consistent with the automatic spreading activation theory, which proposed that the processing of the prime stimuli can activate its representation in the semantic network and the activation can automatically spread to semantically related nodes in the network, resulting in increased activation of their representation. Therefore, the processing of semantically related targets was facilitated (Kiefer, 2002). However, it should be pointed out that the nonsignificant topography comparison did not provide direct evidence that the repetition and conceptual N400 priming effects had the same neural source because of the poor spatial resolution of ERP and the nonunique solution of the inverse problem (Luck, 2014).

As the masked priming paradigm mainly reflects automatic and unconscious processing of the prime stimulus (Forster et al., 2003), results that the N400 was modulated by masked repetition and conceptual priming suggested that the N400 of visual objects was sensitive to automatic semantic spreading. Most previous studies that found the N400 was sensitive to automatic sematic spreading used word as stimuli (Deacon, Hewitt, Yang, & Nagata, 2000; Kiefer, 2002; Kiefer & Spitzer, 2000). To our knowledge, the present study is the first to reveal that the N400 of visual objects is also modulated by the prior masked conceptual prime picture, which extended the evidence supporting the automatic spreading activation explanation of the N400.

In sum, the present study indicates that masked repetition and conceptual priming of visual objects have different time courses of processing. The repetition priming involves both perceptual and semantic processing, which are indexed by the N/P190 and N400 effects, respectively. However, the conceptual priming only involves semantic processing, which is indexed by the N400 effect.

## CONFLICT OF INTEREST

Authors declare no conflict of interest.

## Data Availability

The data of this study are available on reasonable request from the corresponding author.
